# Musical Instrument Practice as Sensorimotor and Motor–Cognitive Skill Training in Healthy Aging

**DOI:** 10.3390/brainsci16101038

**Published:** 2026-09-28

**Authors:** Gianpiero Greco, Domenico Desantis, Stefania Cataldi, Francesco Fischetti

**Affiliations:** 1 Department of Translational Biomedicine and Neuroscience (DiBraiN), University of Bari Aldo Moro, 70124 Bari, BA, Italy; gianpiero.greco@uniba.it (G.G.); francesco.fischetti@uniba.it (F.F.); 2 Flow Scuola di Musica e Teatro, Associazione Movidabilia APS ETS, 74028 Sava, TA, Italy; info@movidabilia.it; 3 Department of Education and Sport Sciences, Pegaso Telematic University, 80143 Naples, NA, Italy

**Keywords:** healthy aging, musical instrument training, motor learning, sensorimotor integration, neuroplasticity

## Abstract

**Highlights:**

**What are the main findings?**

- Instrumental training in older adults shows selective benefits for bimanual coordination, fine-motor control, and timing, whereas broad transfer to gait, balance, and everyday motor function remains unproven.
- Cognitive and neuroplastic effects are selective and heterogeneous, with changes in processing speed, fluency, working memory, brain structure, connectivity, and oscillatory dynamics alongside frequent null findings.

**What are the implications of the main findings?**

- Musical instrument practice may provide an engaging and adaptable form of sensorimotor and motor–cognitive skill learning in healthy aging.
- Future trials should directly quantify skill acquisition, practice dose, retention, and transfer and compare instrument-specific motor demands using standardized outcomes and appropriate active controls.

**Abstract:**

**Background/Objectives:** Playing a musical instrument requires precisely timed movements, bimanual or interlimb coordination, visuomotor mapping, auditory and somatosensory feedback, sequence learning, prediction, and executive regulation. This narrative review synthesizes motor, cognitive, and neuroplastic adaptations associated with active instrumental practice in predominantly healthy adults aged 60 years or older, interpreting practice as a complex sensorimotor and motor–cognitive skill activity. **Methods:** English-language literature from January 2016 through August 2026 was searched in PubMed/MEDLINE, Scopus, and Web of Science Core Collection, supplemented by Google Scholar and citation chasing. Randomized and controlled interventions were prioritized, with longitudinal and observational evidence used contextually; earlier studies were included selectively for seminal or mechanistic context. **Results:** Direct motor evidence was less abundant than cognitive evidence. Both piano and mallet-percussion programs improved task-proximal bimanual synchronization, and one large active-control trial found greater improvement in untrained unimanual fine-motor control after one year of piano practice. Selective near transfer to continuation timing was also reported, whereas evidence for generalized dexterity, gait, balance, lower-limb coordination, and everyday motor function remained limited or mixed. Cognitive effects were selective, particularly for processing speed, verbal fluency or switching, and some working-memory measures. Neuroplastic findings included cortical-thickness increase, relative structural preservation, altered auditory–motor connectivity, reduced task activation, and oscillatory modulation. **Conclusions:** Instrumental practice may provide an engaging and adaptable form of sensorimotor and motor–cognitive skill practice in healthy aging, but broad transfer and neuroprotection are not established. Future trials should quantify skill acquisition, dose, retention, and transfer and compare instrument-specific demands.

## 1. Introduction

Healthy aging depends partly on the capacity to maintain adaptive movement, learn new skills, and coordinate action with changing sensory and cognitive demands. Advancing age is associated with slower movements, greater variability, reduced manual dexterity, less stable interlimb coordination, and changes in sensory integration and motor-network recruitment [1,2]. These changes may interact with declines in processing speed, working memory, and executive control because many everyday actions require concurrent planning, monitoring, and correction rather than isolated muscle output. Recent evidence also indicates that executive functioning, particularly working-memory updating, contributes to complex bimanual motor performance in older adults [3]. Activities that challenge movement and cognition together are therefore of particular interest within movement sciences, provided that their motor demands and transfer effects are directly assessed rather than inferred.

Learning to play a musical instrument is one such activity. Instrumental performance transforms visual, auditory, and proprioceptive information into temporally organized actions [4,5]. Even beginner practice can require finger individuation, graded force, movement sequencing, bimanual coordination, synchronization to an external pulse, error detection, and repeated updating of action plans [6,7,8]. The particular configuration depends on the instrument and curriculum: two-handed keyboard playing differs from one-handed keyboard-harmonica practice, mallet percussion, electronic drumming, bowed strings, or wind performance [6,9,10]. Across modalities, however, instrumental practice is best conceptualized as a complex sensorimotor and motor–cognitive skill activity in which repeated movement is organized by perception, prediction, feedback, and learning.

This distinction matters conceptually and scientifically. Instrumental practice has often been discussed primarily in terms of cognitive enrichment, music-based interventions, or arts interventions. Such perspectives capture important features but may obscure the motor-learning processes that make playing an instrument different from listening to music. Conversely, describing instrument learning as exercise risks implying cardiorespiratory, strength, balance, or osteogenic adaptations that most instrumental-training protocols do not provide. Instrumental practice may complement aerobic, resistance, balance, and multicomponent exercise, but it should not be presented as a substitute for them or for guideline-based physical activity [11].

The empirical literature has expanded over the past decade, including randomized comparisons of piano with active musical controls, direct contrasts between fine- and gross-motor instrumental curricula, short-term piano studies of temporal processing, keyboard-harmonica interventions, longitudinal retention studies, and structural and functional or electrophysiological studies [6,8,9,12,13,14]. The field nevertheless remains difficult to interpret. Motor outcomes are less frequently measured than cognition; active musical comparators can produce shared benefits; several publications derive from the same parent trials; and neural findings are heterogeneous, encompassing structural increase, relative preservation, reduced task activation, altered connectivity, and oscillatory modulation rather than a single uniform form of “neuroplasticity.”

A 2024 systematic review and meta-analysis focused on fluid intelligence and executive functions in healthy older instrument novices and reported small-to-moderate effects for inhibition, switching, verbal category switching, and processing speed, but no pooled effects for selective visual attention, working memory, or verbal memory; most included studies were rated as having some concerns or high risk of bias [15]. Building on, but extending beyond, this cognition-centered synthesis, the present review interprets instrumental practice explicitly as complex sensorimotor and motor–cognitive skill training and integrates motor function, motor learning, instrument-specific demands, cognitive transfer, and neuroplastic findings within a movement-centered framework. The primary aims are to synthesize the motor and sensorimotor adaptations directly assessed in healthy older adults, evaluate the selectivity and specificity of cognitive findings, distinguish among different types of neural adaptation, examine instrument-specific motor demands and training characteristics, and identify methodological priorities for testing motor learning, retention, and transfer in healthy aging.

## 2. Materials and Methods

### 2.1. Review Design

This article is a narrative review. A narrative design was selected because the available evidence is heterogeneous with respect to instrument type, training duration and dose, comparator conditions, motor and cognitive outcomes, and neuroimaging or neurophysiological methods, making a single quantitative synthesis inappropriate for the aims of the present review. Its methods were designed to make the literature search, eligibility decisions, and synthesis traceable without implying the exhaustiveness or formal certainty assessment of a systematic review. Methodological guidance for transparent narrative reviewing informed the approach [16,17]. No meta-analysis, formal risk-of-bias assessment, or certainty-of-evidence grading was undertaken.

### 2.2. Literature Search

The principal search covered English-language publications from 1 January 2016 through the final update on 31 August 2026. PubMed/MEDLINE, Scopus, and Web of Science Core Collection were searched using combinations of terms for instrumental modalities, older adults, motor and sensorimotor function, cognition, and neuroplasticity. The final database searches retrieved 510 records from PubMed/MEDLINE, 1274 from Scopus, and 1760 from Web of Science Core Collection. Search terms were organized around four main concepts: active musical instrument practice or training; older adulthood and aging; motor and sensorimotor function; and cognitive or neuroplastic outcomes. Instrument-related terms included combinations such as “musical instrument,” “musical instruments,” “instrumental practice,” “instrumental training,” “music training,” “musical training,” “instrument playing,” “instrument learning,” piano, keyboard, percussion, drum*, drumming, melodica, “keyboard harmonica,” “string instrument,” “string instruments,” violin*, viola, cello, guitar*, “wind instrument,” “wind instruments,” “brass instrument,” “brass instruments,” flute*, clarinet*, saxophone*, recorder*, trumpet*, and trombone*, combined with aging- and outcome-related terms using Boolean operators adapted to the indexing requirements of each database. Google Scholar was used as a supplementary source to identify additional relevant records and support citation tracking. Backward and forward citation chasing was conducted from eligible primary studies and relevant reviews, including Rogers and Metzler-Baddeley [15] and Olszewska et al. [18]. Publications preceding 2016 were not part of the core contemporary evidence synthesis but were cited selectively when they provided seminal evidence, mechanistic context, or essential background concerning motor learning, sensorimotor synchronization, or auditory–motor coupling. Complete database-specific strings are reported in Supplementary Table S1.

### 2.3. Eligibility and Scope

The core population was healthy or cognitively unimpaired older adults, predominantly aged 60 years or older. For the core intervention evidence, priority was given to studies explicitly investigating older-adult populations in which participants were predominantly aged ≥ 60 years. Studies that included younger participants below this threshold were retained only as contextual evidence when the sample was explicitly defined as an older-adult cohort or when findings relevant to participants aged ≥ 60 years could be interpreted appropriately. The principal exposure was active practice of a musical instrument, including piano or electronic keyboard, mallet percussion or drumming, melodica or keyboard harmonica, string and wind instruments, and mixed instrumental ensembles. To contribute to the core synthesis, primary studies were required to examine active instrumental practice and report at least one relevant motor or sensorimotor, cognitive, or neuroplastic outcome. Randomized controlled trials, controlled interventions, quasi-experimental studies, and longitudinal training studies received priority. Observational studies of lifelong or current instrumentalists and longitudinal follow-up studies were included selectively when they contributed direct evidence on motor performance, motor expertise, cognition, brain structure, or brain function.

Studies centered on passive music listening, singing alone, dance, or instrument-based rehabilitation in clinical populations rather than healthy aging were excluded from the core synthesis. Studies primarily involving dementia, major neurological disease, or psychiatric illness were likewise excluded. Mixed healthy and mild-cognitive-impairment samples were retained only as contextual evidence when the healthy subgroup could not be isolated, and this limitation was explicitly identified in the synthesis. Studies of young or professional musicians were not treated as primary aging evidence; selected reports were used only to explain instrument-specific motor demands or plausible auditory–motor mechanisms. Systematic reviews and meta-analyses were used for orientation, study identification, and comparison with prior conclusions, but were not treated as primary evidence in the narrative synthesis. Conference abstracts, editorials, dissertations, protocols, and non-peer-reviewed preprints were not treated as primary outcome evidence.

### 2.4. Study Selection and Data Organization

Potentially relevant records were first screened by title and abstract. Reports considered relevant to the review aims then underwent full-text assessment to confirm eligibility and determine their role within the core or contextual evidence base.

The three database searches retrieved 3544 records before between-database deduplication: 510 from PubMed/MEDLINE, 1274 from Scopus, and 1760 from Web of Science Core Collection. After screening, eligibility assessment, and reconciliation of overlapping publications and parent cohorts, 40 empirical outcome reports were retained for the contemporary evidence synthesis. These comprised 24 core intervention reports representing 13 independent intervention studies or parent trials and 16 contextual or mechanistic reports represented in 13 contextual study/cohort entries. Because the four-year follow-up by Wang et al. [19] derived from a parent cohort already represented in the core intervention evidence, the retained empirical evidence corresponded to 25 independent parent studies or cohorts overall. James et al. [20] was retained separately as the protocol for the Train the Brain with Music trial and was not counted as an outcome report.

For each retained empirical report, information was organized on country, design, sample and age, previous musical experience, instrument and curriculum, supervised and home-practice dose, comparator, motor or sensorimotor outcomes, cognitive outcomes, neuroplastic outcomes, follow-up, and principal findings. Claims in the synthesis were checked against the original report, with particular attention to between-group interactions, within-group change, the interpretation of multiple outcomes, and whether imaging effects represented increase, stability, decline, reduced activation, or altered connectivity.

When multiple publications originated from the same parent trial or cohort, the reports were linked and treated as deriving from a single independent participant sample rather than as independent replications. This approach was applied consistently across the evidence tables and narrative synthesis to reduce the risk of overestimating replication when one cohort generated several publications addressing different behavioral or neuroplastic outcomes.

### 2.5. Narrative Synthesis

Evidence was synthesized thematically in the following order: the sensorimotor and motor–cognitive framework; motor and sensorimotor outcomes; cognitive outcomes; structural and functional neuroplasticity; instrument-specific demands; dose and moderating factors; and methodological priorities for future research. Interpretive weight was based on design, comparator, outcome specificity, sample independence, and consistency across reports. Randomized active-control findings were given greater inferential weight than uncontrolled change, self-selected continuation, or cross-sectional musician–nonmusician differences. Because interventions, measures, and parent cohorts were heterogeneous, numerical pooling was not undertaken.

## 3. Musical Instrument Practice as Sensorimotor and Motor–Cognitive Training

The core contemporary evidence base comprised intervention studies with heterogeneous designs, instrumental modalities, training doses, comparators, and outcome domains. The main characteristics of the core intervention studies contributing to the narrative synthesis are summarized in Table 1. Selected mixed-status, multicomponent, longitudinal follow-up, and observational studies that informed contextual or mechanistic interpretation are reported separately in Supplementary Table S2. Publications derived from the same parent trial or cohort are grouped accordingly to avoid overestimating the number of independent participant samples.

### 3.1. Perception–Action Loops in Instrumental Performance

Instrumental practice is organized around recurrent perception–action loops. A performer selects or reads a musical target, predicts its sensory consequences, generates a movement, compares auditory and somatosensory feedback with the intended outcome, and adjusts the next action. The loop can operate at multiple time scales, from correcting a mistimed keystroke to reorganizing an entire phrase. Auditory–motor interactions are central to both perception and production of music, while premotor, parietal, cerebellar, basal ganglia, and auditory systems contribute differently to sequencing, prediction, timing, and error correction [4,5].

Visual demands vary with the task. Reading notation requires mapping symbolic spatial information onto effectors and locations; watching an instructor adds action observation; looking at the hands or keys supplies online visuomotor information. Somatosensory and proprioceptive feedback report key resistance, contact timing, joint configuration, mallet rebound, bow position, or airflow-related pressure, depending on the instrument. Auditory feedback provides an external consequence of movement that is unusually immediate and information-rich. These combined signals make instrumental practice more than repetitive finger movement, but they also complicate causal attribution because perceptual, cognitive, social, and motor ingredients change together [4,5].

### 3.2. Fine-Motor Control, Coordination, and Timing

Keyboard performance commonly trains individuated finger actions, graded timing and force, and bimanual coordination. The hands may act in parallel, alternate, or perform different rhythmic and melodic roles. Mallet percussion can emphasize larger proximal movements, spatial targeting, rebound control, and rhythmic synchronization, although some mallet tasks also demand fine grip and distal control [6]. Percussion-based improvisation can impose a different motor profile: in Santos et al. [32], participants explored and improvised with multiple easy-to-handle percussion instruments, making the motor exposure less standardized and less specifically targeted to finger individuation or bilateral technical skill than the mallet-percussion curriculum described by Bugos [6]. Electronic drumming may involve coordinated rhythmic actions across the upper limbs and, depending on the configuration, the lower limbs, thereby increasing interlimb coordination and postural demands. Melodica combines key selection with respiratory control, but the reviewed older-adult protocols used simplified right-hand performance and therefore imposed less bimanual demand than most piano curricula [9,35]. Expert performance research illustrates how long-term practice can be associated with instrument-specific sensorimotor representations, as in asymmetric hand maps among string players, but such evidence does not establish effects of late-life beginner training [38].

Timing is a cross-cutting requirement. Sensorimotor synchronization involves aligning movement onsets with predictable auditory events, while rhythmic production also requires interval representation, phase correction, and anticipation [7]. In ensemble contexts, performers coordinate with other people and must adjust to timing deviations that are not entirely under their control. These processes are plausible targets for training, yet only a minority of aging trials have used validated timing or synchronization outcomes outside the practiced repertoire.

### 3.3. Motor Learning and Cognitive Control

Instrument learning requires acquisition, consolidation, retention, and flexible retrieval of action sequences. Early practice is attention-demanding and error-prone; with experience, selected components may become faster, more stable, or less dependent on conscious control. Older adults retain substantial motor-learning capacity, although age-related differences may emerge in skill acquisition, consolidation, retention, and transfer compared with younger adults [39]. Training-related change should therefore be evaluated not only by successful completion of the practiced curriculum, but also through changes in speed–accuracy trade-offs, coordination, retention, and performance under modified or untrained conditions.

Executive functions are embedded in this learning process. Attention selects relevant notation, sounds, and bodily feedback; working memory maintains short sequences and task rules; inhibition suppresses premature or incorrect actions; cognitive flexibility supports switching among patterns, hands, or expressive goals; and monitoring processes compare predicted and obtained outcomes [3,4]. These demands make cognitive transfer plausible, but shared task requirements do not guarantee far transfer. Improvement on an untrained cognitive task is therefore more plausible when training and transfer tasks share relevant underlying processes and when training dose, challenge, and outcome sensitivity are sufficient to detect change.

**Figure 1:**
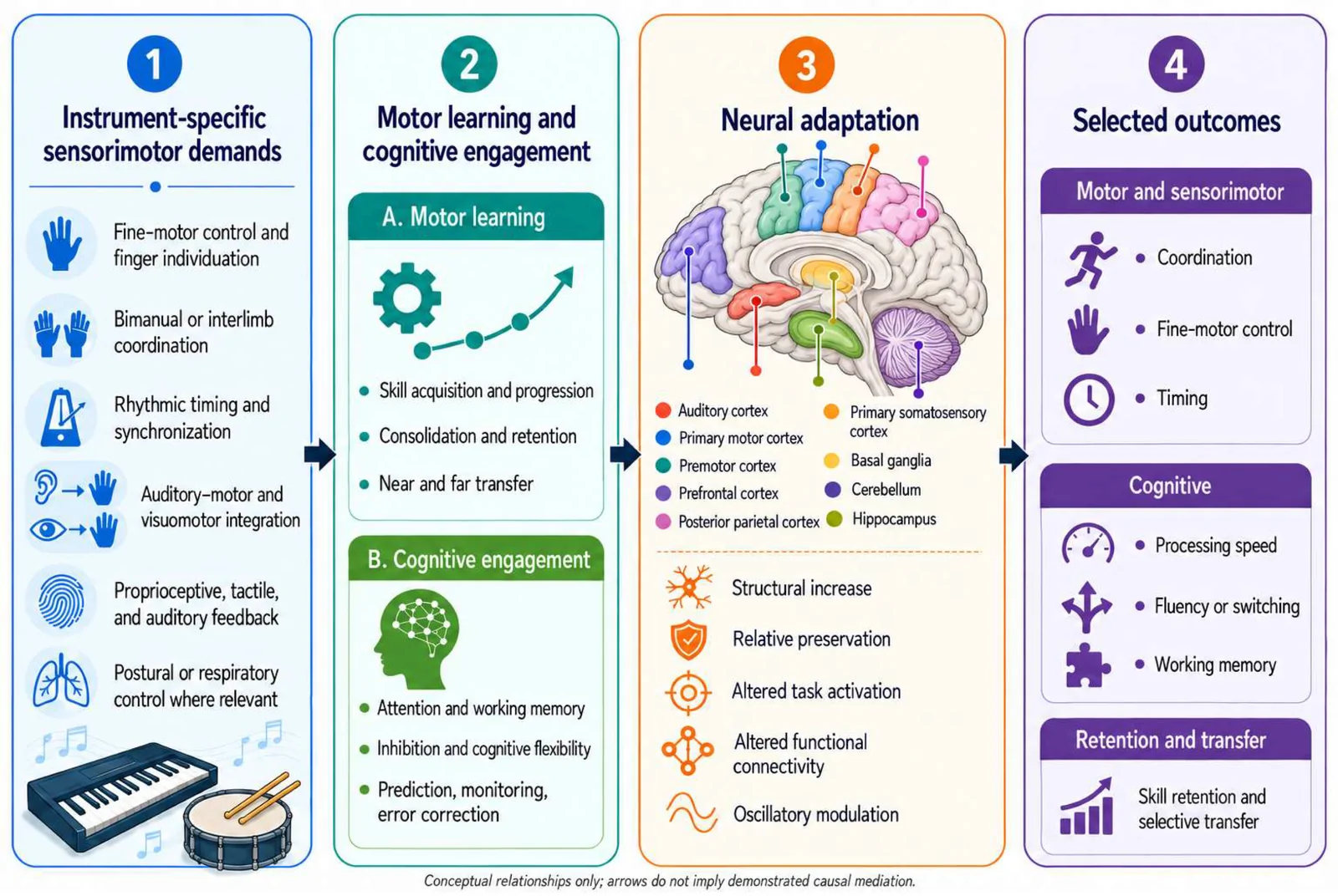
Conceptual framework for interpreting musical instrument practice in healthy aging. Instrumental practice combines fine and, in some modalities, gross-motor actions; bimanual or interlimb coordination; rhythmic timing; auditory–motor and visuomotor integration; and proprioceptive, tactile, and auditory feedback. These demands occur in conjunction with attention, executive control, working memory, prediction, and error monitoring. Repeated practice may support motor-skill acquisition, consolidation, retention, and potential transfer while engaging auditory, premotor, primary motor, primary somatosensory, posterior parietal, cerebellar, basal ganglia, prefrontal, and hippocampal systems. Neural adaptations may include structural increase, relative preservation, altered task activation, altered functional connectivity, or oscillatory modulation, and may accompany selected motor and cognitive outcomes. *Note.* Arrows depict conceptual temporal and functional relationships and do not imply causal mediation. The reviewed studies rarely tested complete indirect pathways. Fine motor refers to small, precisely controlled movements; gross motor refers to larger proximal or multilimbed actions. The brain illustration is schematic; highlighted regions indicate the principal neural systems represented in the reviewed evidence and do not imply uniform training-induced effects or exhaustive anatomical coverage. *Source:* Authors’ own elaboration. The initial graphical rendering was generated using the OpenAI ChatGPT Images 2.0 generation tool and subsequently reviewed and refined by the authors. All scientific content and graphical elements were verified by the authors for accuracy and completeness.

Figure 1 summarizes the movement-centered framework used in this review. It links instrument-specific motor and sensorimotor demands with cognitive engagement, motor learning, neural adaptation, and possible behavioral outcomes without assuming that each intermediate pathway has been causally tested.

## 4. Effects of Instrumental Practice on Motor and Sensorimotor Function

### 4.1. Extent and Characteristics of the Motor Evidence Base

Motor and sensorimotor outcomes are conceptually primary for a movement-centered review, but they are not the most frequently measured outcomes in the literature. Many trials test executive function or memory while treating motor practice as the intervention ingredient rather than an outcome. This produces an evidentiary asymmetry: the field often invokes finger dexterity, bimanual coordination, and timing as mechanisms without establishing that these capacities changed outside the trained musical task.

Direct evidence comes primarily from protocols that measured coordination or fine-motor control before and after training. Among 135 completers in a matched three-arm study of healthy adults aged 60–80 years, piano, mallet-percussion, and music-listening groups completed 16 weeks of instruction with equivalent class duration and assigned homework [6]. Compared with music-listening instruction, both piano and percussion training improved bimanual synchronization, with pairwise comparisons showing the largest synchronization gain after piano training. Because the outcome resembled the coordinated musical actions practiced in class, this finding supports task-proximal learning or near transfer more strongly than generalized manual improvement. The nonrandom allocation and 25% overall attrition also limit causal precision.

The Train the Brain with Music randomized trial provides particularly informative longer-term evidence of transfer to untrained fine-motor tasks. Across one year, piano practice produced greater improvement than music-culture lessons on measures of unimanual fine-motor control, with individual trajectories becoming most distinguishable during the second six months [8]. Within the piano group, change in unimanual performance co-developed with gray-matter change in contralateral primary motor cortex during that later interval. This result is consistent with motor-skill consolidation in sensorimotor networks, but the latent coupling analysis did not establish that cortical change mediated the behavioral effect.

### 4.2. Dexterity, Finger Individuation, and Movement Speed

Evidence for generalized finger dexterity is mixed. Bugos [6] did not report a piano-specific advantage on simple finger-tapping speed despite the advantage in bimanual synchronization. MacRitchie et al. [34] assessed domain-general motor function using the Jebsen Taylor Hand Function Test and a visuomotor synchronization task but did not observe the hypothesized gains in these measures. Bayesian modeling nevertheless provided moderate evidence for improvement on Trail Making Test Part A, which the authors interpreted as a visuomotor effect. The same analyses provided moderate evidence for negative training effects on Part B and the B–A difference score, suggesting no benefit for cognitive switching. Given the very small sample, these mixed findings should not be interpreted as evidence of generalized manual-dexterity improvement.

A randomized active-comparator study provides an additional direct test of motor transfer from percussion-based practice. After eight weeks of percussion and musical improvisation, no significant group-by-time effects were observed for Timed Up and Go performance or for right- or left-hand finger tapping compared with choir training [32]. Because the intervention involved active percussion practice but did not improve these untrained motor outcomes, this null finding cautions against inferring generalized motor transfer from the motor demands of instrumental practice alone.

The twelve-month Active Minds Music Ensemble offers a more detailed account of skill acquisition. Sixty-eight novice older adults learned keyboard-based tasks online, alternating a digital piano and a touchscreen virtual keyboard and practicing melodic replication and improvisation in a counterbalanced longitudinal design [12,37]. Musical accuracy and selected keyboard skills improved over time and were retained at six-month follow-up [12]. In the related motor analysis, dual-finger tapping improved across repeated assessments, whereas maximal single-finger tapping did not [37]. This dissociation is informative: training may affect coordination or task organization without increasing elementary movement speed. Because the study lacked an external control, repeated testing and other nonspecific longitudinal effects cannot be excluded, although instrument order was randomized and counterbalanced.

### 4.3. Bimanual and Interlimb Coordination

Bimanual coordination is one of the most plausible near-transfer targets because it is explicitly practiced in piano and many percussion curricula. The Bugos [6] comparison suggests that fine-motor keyboard practice and gross-motor mallet practice can both change coordinated performance, while the larger piano-specific synchronization effect may reflect greater similarity between the training and test. This interpretation is preferable to the broader claim that fine-motor practice is inherently superior. Percussion curricula can emphasize different effectors, spatial amplitudes, and phase relationships, and the selected outcome may favor one modality.

Evidence for interlimb coordination involving the lower limbs is minimal. Shinada et al. [10] allowed novices to select keyboard, electronic drums, or bass guitar and rehearse together for 16 weeks, thereby introducing heterogeneous bilateral and, for some instruments, hand–foot coordination demands. However, the study assessed cognition and mood rather than gait, balance, postural control, or limb coordination. Similarly, rhythm–motor dual-task interventions have sometimes combined walking, instrument playing, and tapping, but these protocols are adjacent to instrumental skill learning and cannot isolate the contribution of learning an instrument [40].

### 4.4. Rhythmic Timing and Sensorimotor Synchronization

Rhythmic accuracy improved in the active instrumental conditions reported by Bugos [6], whereas the most specific between-group effect concerned synchronization in the piano group. The result cautions against treating every timing-related effect as motor-specific. Listening assignments can train temporal attention and explicit rhythmic knowledge, and repeated exposure to the test can improve performance. Direct contrasts using standardized synchronization tasks, untrained tempi, and perturbation paradigms would be more informative than pre–post performance of practiced patterns.

A small nonrandomized controlled study provides rare evidence of near transfer to temporal processing outside the practiced piano repertoire. After approximately one month of individual piano training, older novices showed improved continuation-phase finger-tapping performance relative to participants who received no training [13]. The reported behavioral improvement concerned internally maintained continuation timing rather than a broad enhancement across all components of sensorimotor synchronization. This finding therefore supports selective near transfer to internal timing representations rather than generalized rhythmic improvement.

The Active Minds data similarly show that melodic learning and melodic discrimination improved more clearly than beat detection or rhythmic precision in a curriculum centered on keyboard melody [12]. This does not imply that older adults cannot improve rhythmic timing; it indicates that the practiced dose and assessment must target it. Evidence from percussion remains limited: one larger matched comparison and one small randomized active-comparator improvisation study are available, whereas other percussion studies are small or combine drumming with singing or additional activities. The independent effect of gross-motor rhythmic practice therefore remains uncertain.

A 10-week melodica trial included a paced-continuation tapping task intended to index rhythm entrainment and auditory–motor transformation [35]. The coefficient of variation did not improve, whereas phonological fluency changed and the working-memory reaction-time result was marginal after false-discovery-rate correction. A Grooved Pegboard measure was administered only as a screening or filler measure and was not included in the outcome analysis. Thus, the study provides a direct null finding for the timing task while also illustrating the importance of distinguishing administered measures from prespecified analyzed outcomes.

### 4.5. Visuomotor, Auditory–Motor, and Sensory Integration

Instrumental practice continually couples movements to visual and auditory consequences, but transfer measures of auditory–motor and visuomotor integration are uncommon. Piano-related improvement on Trail Making Test Part A may involve visual scanning and graphomotor speed, but it does not isolate visuomotor adaptation [34]. In Train the Brain with Music, speech-in-noise perception improved in both piano and music-culture groups under binaural presentation; piano-specific differences were lateralized, varied by site, and appeared predominantly in women [21]. This pattern does not support an undifferentiated auditory-transfer claim. Structural and functional neural findings provide complementary evidence of auditory-system adaptation, but these measures should not be treated as substitutes for behavioral measures of sensorimotor integration [22,25].

The large Keys to Staying Sharp trial is especially important because it tested whether piano training outperformed an active music-listening curriculum on auditory processing, cognition, and everyday function in 268 older adults with and without mild cognitive impairment [41]. Piano showed no superiority on any prespecified outcome. The mixed cognitive-status sample falls outside the healthy-only core of this review, but the result shows that, in this trial, adding active piano training did not produce measurable transfer beyond an active music-listening comparator.

### 4.6. Retention and Motor Transfer

Retention has rarely been measured with motor outcomes after a no-practice interval. The Active Minds cohort retained several musical skills six months after training, demonstrating durable retention of practiced musical material in late-life novices [12]. The Train the Brain with Music trial included an 18-month follow-up, but published motor analyses have concentrated mainly on change during the intervention. Other reports use immediate posttests or short follow-up intervals and do not distinguish temporary performance effects from durable learning.

Taken together, the motor evidence supports the trainability of musical coordination, selected fine-motor functions, and some aspects of internally maintained timing in older adults. It does not yet establish broad transfer to everyday manual dexterity, gait, balance, or fall-related function. Future work should separate task-specific acquisition, near transfer to structurally similar motor tasks, and far transfer to daily activities, and should report retention and speed–accuracy functions rather than endpoint speed alone.

## 5. Effects on Cognitive Function

### 5.1. Executive Control and Processing Speed

Cognitive findings are more numerous than motor findings but are selective. A two-week intensive piano program was associated with improved processing speed and verbal fluency relative to a preceding no-training period, while verbal memory did not improve [30]. The within-person control period and small sample provide only limited protection against expectancy, retest, and instructor effects.

In the larger 16-week comparison by Bugos [6], piano and percussion improved visual scanning and working-memory performance relative to music listening, whereas response inhibition did not change. Processing speed and verbal fluency improved over time, but the specificity of these changes was limited. A small randomized active-comparator study of percussion-based musical improvisation provides a complementary result. After eight weekly sessions, a group-by-time interaction favored percussion/improvisation over choir training on the Clock Drawing Test, whereas Trail Making Test Part A improved across groups and the remaining executive measures did not show comparable intervention-specific effects [32]. Given the small sample and limited duration, this finding supports selective rather than broad cognitive transfer.

A separate randomized trial of 155 adults aged 60–80 years compared piano, computerized cognitive training, and no treatment [29]. Both active conditions improved working memory and processing speed relative to no treatment, while piano showed a more specific advantage on category-switching verbal fluency. This pattern supports selective transfer but also shows that some benefits are shared with a nonmusical cognitive intervention.

Jazz-piano training provides preliminary evidence that improvisational demands may engage executive control. In a pilot randomized study, 37 completers underwent an intensive two-week jazz-piano program or no treatment [33]. Training improved Paced Auditory Serial Addition Task performance and reduced Stroop errors in the mixed condition relative to control, whereas no significant training effect was found for overall verbal fluency. Electroencephalographic results are discussed below. The concentrated dose, small sample, and passive comparator mean that novelty, social contact, and expectation remain alternative explanations.

Longer training does not produce uniform executive benefit. In the Train the Brain with Music cohort, both piano and music-culture groups improved on several cognitive-flexibility measures over one year; the piano group showed a larger improvement only for number-switch mixing cost, and change in pianistic skill was not associated with change in cognitive flexibility [28]. In a 16-week mixed-instrument band trial, significant group-by-time interactions were observed for global cognition, assessed with the Mini-Mental State Examination (MMSE), and delayed logical memory, whereas Trail Making, Stroop, digit-span, and verbal-fluency measures did not show comparable intervention effects [10]. The result argues against describing the intervention as broadly enhancing executive function.

### 5.2. Working Memory, Verbal Memory, and Fluency

Keyboard-harmonica and melodica studies have yielded domain-specific memory findings. A 16-week right-hand keyboard-harmonica program improved delayed story recall relative to a passive control but did not produce broad executive or processing-speed gains [9]. A separate 10-week melodica cohort showed a significant advantage in phonological verbal fluency and only marginal evidence for faster Sternberg working-memory responses after correction for multiple testing [35]. A later analysis of that same cohort reported an executive-function composite advantage and found that early music-performance skill predicted cognitive improvement [36]. This association is consistent with a link between successful instrumental skill acquisition and transfer, but it does not establish that motor learning mediated cognitive change.

At four-year follow-up of the 16-week keyboard-harmonica parent cohort, 13 participants who voluntarily continued playing were compared with 19 who stopped [19]. Continued players showed better preservation of a verbal working-memory composite. Because continuation was self-selected after the randomized phase, this finding is longitudinal but not randomized; motivation, health, lifestyle, and initial response could influence both continued practice and outcome.

In the Train the Brain with Music cohort, tonal working memory improved across both intervention groups after six months, with no evidence that this improvement was specific to piano practice [26]. A separate 12-month analysis reported a greater improvement in backward digit span in the piano group than in the music-culture group [8]. These findings illustrate the importance of distinguishing cognitive changes shared by both music interventions from effects that specifically favor active instrumental practice. Shared improvement may arise from auditory analysis, musical knowledge, expectancy, social engagement, or retesting, whereas between-group differences provide a more informative estimate of the incremental effect associated with instrumental practice.

### 5.3. Null Findings, Active Controls, and Specificity

The most informative test of instrument-specific cognitive transfer is often the contrast with an active condition that matches instructor contact, group participation, musical attention, and expectancy. Such comparisons can yield more conservative findings than passive controls. Keys to Staying Sharp found no piano advantage over music listening across auditory-processing, cognitive, or everyday-function measures [41]. Train the Brain with Music shows a mixture of piano-specific effects, shared musical effects, and null differences. The percussion/improvisation trial likewise produced one selective cognitive interaction alongside several null or nonspecific findings when compared with choir training [32]. Similarly, Alain et al. [42] reported limited behavioral specificity across active arts conditions, while several electrophysiological changes were shared between music and visual-art training.

These patterns explain why conclusions differ across reviews. Rogers and Metzler-Baddeley [15] reported statistically significant low-to-moderate effects for switching, inhibition, category-switching verbal fluency, and processing speed, but no significant pooled effects for working-memory capacity or updating, other verbal-fluency measures, visuospatial attention, or verbal memory, alongside substantial risk-of-bias concerns. The present movement-centered synthesis agrees that cognitive transfer is plausible and selective but emphasizes that its mechanism cannot be inferred merely from the motor richness of the intervention. Stronger attribution of cognitive transfer to the motor-learning component of instrumental practice would require concurrent evidence of motor-learning and cognitive change, an appropriate active comparator, and, ideally, prespecified mediation or process analyses.

### 5.4. Observational Evidence and Cognitive Reserve

Observational studies broaden the time scale but cannot establish causality. In the Wisconsin Longitudinal Study, musical engagement in adolescence and continued adult instrument playing were associated with higher episodic-memory level around age 65, but continued adult playing was not associated with a slower subsequent rate of decline [43]. A matched DELCODE analysis found better global cognition, working memory, executive function, language, and visuospatial performance among adults reporting instrumental activity across life, but no group difference in learning and memory [44]. Lifelong musicians have also shown different executive-control event-related potentials and selected behavioral advantages [45].

Such results are compatible with cognitive reserve, maintenance of cognitive function, or selection into sustained musical activity. They do not determine whether music caused the group difference. Education, socioeconomic resources, hearing, health, personality, prior cognition, and aptitude can affect both continued musicianship and late-life performance. Observational evidence is therefore best used to motivate longitudinal trials and identify candidate outcomes, not to claim prevention of cognitive decline.

## 6. Neuroplastic Adaptations Associated with Instrumental Training

### 6.1. Structural Increase Versus Relative Preservation

Structural magnetic resonance imaging (MRI) findings require precise language. In the Train the Brain with Music trial, six months of piano training produced greater cortical-thickness increases than music-culture lessons in five auditory-related regions: left Heschl’s gyrus, left planum polare, bilateral superior temporal sulcus, and right Heschl’s sulcus [22]. This is evidence of regionally specific cortical-thickness increase relative to an active comparator. In contrast, fixel-based analysis showed declining fornix fiber density in the music-culture group and stability in the piano group [14]. The latter is relative preservation or stabilization, not an increase in white matter. Changes in fornix fiber density were also associated with episodic-memory performance, providing a brain–behavior correlate without establishing causality.

Voxel-based morphometry in 132 participants found gray-matter increases in the caudate, Rolandic operculum, and inferior cerebellum when both intervention groups were pooled, with no between-group difference in these clusters [26]. These changes therefore support plasticity associated with musical education or shared intervention factors rather than a piano-specific motor effect. A region-of-interest analysis indicated that right primary auditory-cortex gray matter declined in the music-culture group while remaining stable in the piano group, again indicating relative preservation. Cerebellar gray-matter change and training intensity were associated with tonal working-memory improvement, but the analysis did not establish mediation.

After one year, there was no piano-specific group-level increase in gray-matter volume of primary motor cortex, putamen, or thalamus. Instead, latent change analyses showed that piano-related improvement in unimanual fine-motor control co-developed with change in contralateral primary motor-cortex gray matter during the second six months of training, while primary motor cortex also showed coordinated change with ipsilateral putamen and thalamus [8]. These coupling patterns are consistent with consolidation in sensorimotor circuits. They represent coordinated individual change rather than generalized growth of motor-related gray matter.

West et al. [46] provide an instructive comparator. In a small randomized study primarily designed to test a three-dimensional video game, the piano group was an active comparison. Hippocampal gray-matter increase occurred in the video-game group rather than the piano group, whereas the passive control showed hippocampal loss. Piano participants showed within-group gray-matter increases in the dorsolateral prefrontal cortex, and both piano and video-game training were associated with cerebellar increases. Given the very small final groups, the age range of 55–75 years, which extends below the principal population threshold of this review, and the study’s primary video-game hypothesis, these findings remain contextual evidence of regional plasticity and require replication.

### 6.2. Functional Connectivity and Task Activation

Functional changes are heterogeneous. After the 16-week keyboard-harmonica program, participants showed reduced activation in right supplementary motor area, left precuneus, and bilateral posterior cingulate regions during a visual working-memory task, together with reduced task-related connectivity between the right posterior cingulate gyrus and left middle temporal gyrus and between the left putamen and right superior temporal gyrus [9]. Greater reduction in left putamen–right superior temporal connectivity was associated with greater memory improvement. The authors interpreted this pattern as improved neural efficiency, but reduced activation alone cannot distinguish efficiency from strategy change, reduced engagement, habituation, vascular factors, or altered task difficulty. The most defensible description is reduced task activation and altered connectivity accompanied by improved delayed verbal memory.

After a full year in Train the Brain with Music, piano practice was associated with increased resting-state connectivity between right Heschl’s gyrus and other regions of the right dorsal auditory stream relative to the music-culture condition [25]. Piano training was also associated with increased connectivity between the right motor hand area and a bilateral predominantly motor network, and this change correlated positively with improvement in fine-motor dexterity. These findings are compatible with training-related reorganization of auditory–motor and motor networks, but they do not imply that all network connectivity increased or that connectivity changes caused behavioral transfer.

The four-year keyboard-harmonica continuation report adds longitudinal but nonrandomized evidence. Continued players showed better preservation of right putamen gray matter, lower cerebellum–pons connectivity related to better verbal working memory, greater cerebellar activation on one task, increased intracerebellar connectivity, and reduced cerebellar–cortical connectivity on another [19]. The mixed direction of these effects is not contradictory; connectivity magnitude is not a generic index of brain health. Because continued practice was self-selected and the follow-up sample was small, the findings should be treated as associations with sustained engagement rather than causal long-term effects of continued instrumental training.

### 6.3. Electrophysiological Adaptations

Electrophysiology can distinguish changes in timing and oscillatory dynamics that structural imaging cannot capture. A small controlled piano-training study provides particularly relevant evidence linking temporal motor learning with electrophysiological adaptation. Following approximately one month of individual piano training, older novices showed altered beat-related beta-band modulation during passive metronome listening, alongside improved continuation-phase finger-tapping performance [13]. Magnetoencephalographic changes involved a broader range of beat-induced beta modulation across auditory, sensorimotor, premotor, parietal, cerebellar, striatal, and medial-prefrontal regions. These findings indicate altered oscillatory dynamics accompanying improvement in internally maintained timing; they do not establish generalized neural enhancement or demonstrate that the neural change mediated the behavioral effect.

Alain et al. [42] found larger auditory N1 and P2 responses to piano tones after both music and visual-art training, with these changes persisting at three-month follow-up. Training-specific effects were also observed during visual response-inhibition processing, with music training producing stronger right-frontal responses related to inhibitory control than visual-art training. Subsequent time–frequency analyses of the same parent cohort provided additional evidence of training-related changes in oscillatory dynamics. Bugos et al. [47] reported increased auditory-evoked theta power after 12 weeks of training in both the music and visual-art groups relative to no-treatment controls, with the increase maintained at three-month follow-up. Because this effect was shared across the two active arts conditions, it supports experience-dependent plasticity more strongly than a music-specific effect. In a separate reanalysis of the same cohort, Lu et al. [48] found that theta activity indexing response suppression during a visual Go/NoGo task was reduced after music training, whereas comparable pre–post change was not observed in the visual-art or no-treatment groups; this change was accompanied by altered functional connectivity among prefrontal, visual, and auditory regions. These findings extend the ERP evidence by showing that training can also modify the temporal and oscillatory organization of neural activity. However, Alain et al. [42], Bugos et al. [47], and Lu et al. [48] derive from the same parent cohort and should not be interpreted as independent replications. The music curriculum also combined body percussion, voice, and non-pitched instruments, limiting attribution to instrumental motor practice.

In the jazz-piano pilot, frontal theta during an improvisation-continuation task and resting parietal alpha showed group-by-time interactions [33]. Frontal theta was maintained in the training group while declining in controls rather than simply increasing after training. At rest, parietal alpha decreased after jazz-piano training while increasing in controls. The appropriate interpretation is modulation or maintenance of oscillatory dynamics rather than generalized enhancement of neural activity or neural efficiency. Observational work in lifelong musicians likewise reports different event-related potentials during executive control, but pre-existing traits and cumulative life experience remain plausible explanations [45].

### 6.4. Observational Neuroimaging

Cross-sectional neuroimaging converges on auditory, sensorimotor, cerebellar, and integrative networks, while also illustrating the limits of causal inference. Older lifelong musicians showed faster tapping, greater bilateral cerebellar gray-matter volume, and stronger cerebellar–hippocampal connectivity than matched nonmusicians; within musicians, tapping speed correlated with executive function [49].

In another shared parent cohort of older adults, with analytic sample sizes varying across reports, greater self-reported musical training was associated with larger inferior-frontal and parahippocampal volumes, and musical sophistication was associated with both greater and lower insula-based connectivity depending on the connection [50,51]. These two articles derive from the same parent cohort and should therefore not be counted as independent evidence; moreover, singing ability contributed substantially to some of the functional-connectivity associations.

The matched DELCODE cohort showed greater gray-matter volume in a somatosensory region among participants with lifelong instrument activity, without hypothesized group differences in higher-order frontal, temporal, or hippocampal regions [44]. These selective positive and null results argue against a unitary “musician brain” effect. Rather, they suggest selective structural associations with long-term musical engagement that require prospective confirmation and should not be interpreted as training-induced neuroplasticity.

## 7. Instrument-Specific Motor Demands and Evidence

### 7.1. Why Modality Matters

“Instrument training” is not a single motor exposure. Instruments vary in effector set, bilateral symmetry, movement amplitude, force control, spatial mapping, respiratory involvement, posture, notation, and feedback. A curriculum can further change the demand by restricting one hand, simplifying rhythm, removing notation, or emphasizing improvisation. Table 2 therefore summarizes the motor and sensorimotor demands of instrument modalities represented in the reviewed evidence, together with major evidence gaps. Entries describe the studied protocols where available and should not be interpreted as universal properties of every instrument within a family.

### 7.2. Piano and Electronic Keyboard

Piano is the most extensively studied modality and includes the largest active-control cohort examining long-term fine-motor and neuroplastic change. Typical reviewed curricula required two-hand key presses, finger sequencing, visual–spatial mapping, notation, temporal precision, and auditory feedback. Some included scales and technical exercises, whereas others used simplified visual notation, computerized instruction, jazz improvisation, or no home practice. These differences matter: a protocol emphasizing chordal accompaniment and improvisation may challenge generative planning differently from one emphasizing scale regularity or score reading.

The evidence supports piano-related improvement in bimanual synchronization, selected untrained fine-motor measures, continuation-phase timing, musical performance, and some executive outcomes [6,8,13,29]. Mechanistic studies of expert performance further illustrate the flexible organization of highly practiced piano movements but cannot establish the effect of beginning in older age [55]. The aging evidence also contains null results, including no piano advantage in the large mixed-status Keys to Staying Sharp trial [41], no uniform effect on simple finger speed, and shared rather than piano-specific cognitive or gray-matter changes in active-control trials. Piano should therefore be regarded as the best-studied modality, not as established as universally superior.

### 7.3. Percussion and Drumming

Mallet percussion and drumming provide an important contrast to keyboard fine-motor practice. The Bugos [6] mallet curriculum required bilateral striking, scale and ostinato patterns, spatial targeting, and rhythmic ensemble coordination. It improved bimanual synchronization and visual scanning and working-memory performance relative to listening, although piano produced a larger synchronization change on the selected task. A smaller mallet study reported improved musical self-efficacy and a trend rather than definitive improvement on cognitive switching relative to autobiographical discussion [31].

A randomized comparison of percussion-based musical improvisation with choir training provides a complementary boundary condition. The improvisation curriculum used multiple easy-to-handle percussion instruments and progressively moved from sound exploration toward structured and free improvisation [32]. Despite this active motor exposure, neither finger-tapping performance nor Timed Up and Go showed an intervention-specific improvement, reinforcing the distinction between instrumental motor engagement and transfer to untrained motor function.

Other percussion evidence mixes instrumental activity with singing, body movement, or non-pitched sound production [42,54]. These programs may be valuable, but they do not isolate the effect of drumming. Electronic drum protocols may impose hand–foot coordination and substantial proximal limb involvement, yet reviewed trials have not measured balance, gait, or lower-limb coordination. The hypothesized advantage of gross-motor or interlimb demand therefore remains largely untested. Similarly, despite the strong bilateral, rhythmic, and auditory–motor demands of percussion and drumming, their instrument-specific neuroplastic effects have not yet been directly investigated in the eligible healthy older-adult training studies identified in this review.

### 7.4. Melodica and Keyboard Harmonica

Melodica or keyboard harmonica combines a small keyboard with exhalation, but the principal Japanese interventions used simplified right-hand performance. Their motor exposure consequently differed from two-handed piano and from wind instruments that demand continuous embouchure control and complex fingering across both hands. The evidence concerns delayed verbal memory, verbal fluency, task activation, connectivity, and selected oral and physical-function measures more than generalized manual or respiratory outcomes [9,19,35,52]. In a small observational 10-month keyboard-harmonica class study, lip oral diadochokinesis improved slightly, whereas grip strength and skeletal muscle mass declined [52]. Because participation was self-selected and training occurred during COVID-19-related movement restrictions, these changes cannot be attributed confidently to the instrument itself. No reviewed controlled trial established improved respiratory function, breath–movement coordination, or generalized finger dexterity. These protocols should not be used to infer effects of the wider wind-instrument family.

### 7.5. Strings, Conventional Wind Instruments, and Mixed Ensembles

String instruments remain strikingly underrepresented in late-life beginner interventions. Mechanistic studies of expert string players demonstrate asymmetric sensorimotor organization consistent with left-hand fingering demands, but they do not answer whether an older novice acquires similar adaptations or functional benefits [38]. Wind-instrument evidence is also sparse, although the updated search identified an uncontrolled 12-week group recorder program in older musical novices [53]. The sample included cognitive performance ranging from normal to mild cognitive impairment and lacked a comparison group, so the study provides contextual evidence of feasibility and cognitive change rather than controlled healthy-aging evidence. No controlled healthy-only late-life beginner intervention was identified for conventional woodwind or brass modalities. Consequently, the distinctive effects of embouchure, respiratory pressure, two-hand fingering, and wind-instrument postural demands remain largely untested.

Mixed electronic ensembles increase ecological validity and allow participant choice, as in the keyboard–drum–bass program of Shinada et al. [10], but they reduce mechanistic specificity unless outcomes are stratified by instrument. Touchscreen keyboards reduce key travel, force, and tactile landmarks relative to digital pianos while preserving spatial selection and auditory feedback; Active Minds showed that both interfaces can support learning within a crossover design in which instrument and task order were randomized and counterbalanced. However, the absence of an external comparator limits causal inference about training-related change [12,37]. These designs highlight why direct instrument comparisons should manipulate motor demand rather than rely solely on family labels.

**Figure 2:**
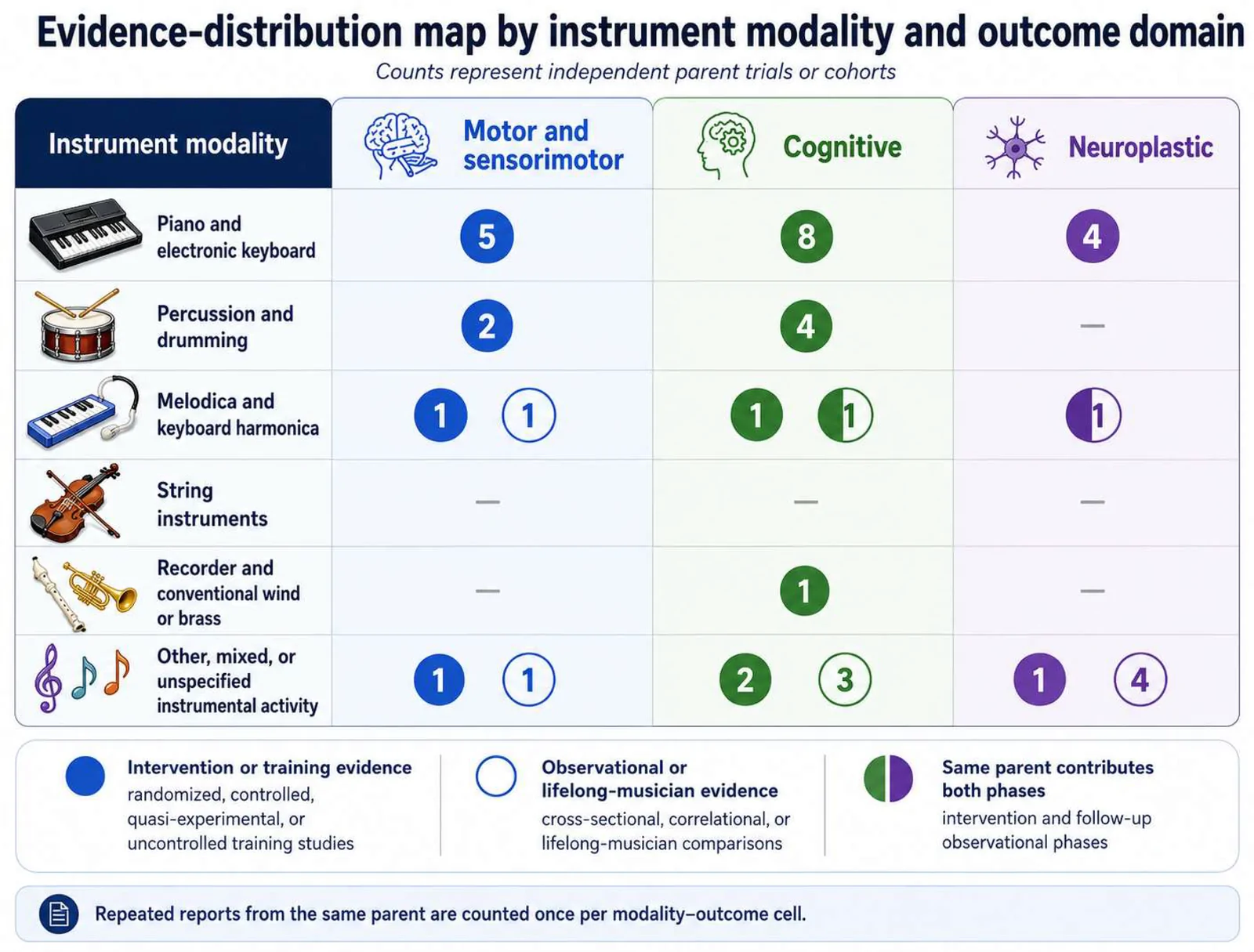
Evidence-distribution map by instrument modality and outcome domain. Rows represent piano and electronic keyboard, percussion and drumming, melodica and keyboard harmonica, string instruments, recorder and conventional wind or brass instruments, and other, mixed, or unspecified instrumental activity. Columns represent motor and sensorimotor, cognitive, and neuroplastic outcomes. A filled circle (●) denotes intervention or training evidence, and an open circle (○) denotes observational or lifelong-musician evidence; the numeral within each symbol indicates the number of independent parent trials or cohorts, not the number of publications. A split symbol denotes a parent cohort contributing both intervention and follow-up observational evidence. Empty cells indicate that no eligible evidence was identified in the contemporary empirical corpus represented in the map; selected pre-2016 seminal or mechanistic sources cited elsewhere in the review are not counted. Symbol size is constant and does not encode effect magnitude, quality, or certainty. *Note.* The map represents evidence of presence and distribution only. It does not constitute a risk-of-bias appraisal, formal certainty-of-evidence assessment, or effect-size display. A parent trial or cohort is counted once per modality–outcome cell even when it generated multiple articles. *Source:* Authors’ own elaboration based on Table 1 and Supplementary Table S2. The graphical rendering was generated using the OpenAI ChatGPT Images 2.0 generation tool and subsequently reviewed and refined by the authors. All study counts, classifications, and scientific content were determined and verified by the authors independently of the AI tool.

Figure 2 summarizes the distribution of evidence across instrument modalities and motor or sensorimotor, cognitive, and neuroplastic outcome domains. The map counts independent parent trials or cohorts and distinguishes intervention from observational evidence without implying formal certainty ratings.

## 8. Training Characteristics, Dose, and Moderating Factors

### 8.1. Duration, Frequency, and Challenge

Reviewed interventions range from 30 concentrated hours over two weeks to weekly lessons sustained for one year. Intermediate-duration protocols further illustrate this heterogeneity, including approximately one month of individualized piano training [13] and eight weekly 60 min sessions of percussion-based musical improvisation [32]. Short intensive piano programs have reported changes in processing speed, fluency, inhibition, and electroencephalographic dynamics [30,33]. Longer programs have shown progressive musical-skill acquisition, selected fine-motor change, cognitive-flexibility change, and structural or functional-connectivity adaptations [8,14,25,27,28]. These observations do not define a dose–response relation because dose covaries with curriculum, comparator, sample, outcome, and analysis. The intensive studies delivered 30 h within two weeks, whereas Train the Brain with Music extended weekly training and home assignments across 12 months.

Weekly contact hours alone underestimate exposure when home practice is assigned. Bugos [6] required approximately three hours per week and used practice records; Train the Brain with Music prescribed approximately 30 min of homework per day on five days per week; Bugos and Wang [29] deliberately omitted outside practice; Shinada et al. [10] prohibited it; and the melodica trial monitored home practice using participant diaries [35]. Practice quantity is therefore measured inconsistently, while practice quality, challenge, errors, rest spacing, and feedback are rarely captured objectively.

From a motor-learning perspective, progression should maintain sufficient challenge to require adaptation without making performance so difficult that persistent errors, frustration, or dropout dominate. Simplified notation, familiar repertoire, tempo adjustment, and instrument choice were used across reviewed protocols to support progression and accessibility. They can also alter visuomotor and executive demands, so adherence-oriented adaptations should be reported as active ingredients rather than treated as neutral delivery details. For example, Train the Brain with Music progressively introduced notation and later increased performance complexity, while its piano-performance assessment introduced a more difficult piece to reduce anticipated ceiling effects [27].

### 8.2. Skill Acquisition as a Manipulation Check

A trial of instrument learning should establish that participants learned the instrument. Several earlier studies provided limited standardized documentation of musical skill acquisition beyond participation, curriculum completion, or proximal training measures. More recent studies have incorporated Musical Instrument Digital Interface (MIDI)-based scale analysis, systematically rated piano performances, computational measures of melodic replication and improvisation, and structured music-performance evaluations [12,27,36]. These approaches document whether the intended instrumental skill was actually acquired and, where follow-up is available, retained. They can serve as manipulation checks and potential moderators. Train the Brain with Music, for example, used MIDI Scale Analysis and systematically rated piano recordings, whereas Active Minds quantified replication and improvisation from MIDI recordings and demonstrated retention over six months.

They should not automatically be treated as mediators. In the 10-week melodica cohort, early performance predicted improvement in an executive-function composite [36], whereas change in pianistic performance was not associated with cognitive-flexibility change in Train the Brain with Music [28]. This inconsistency may reflect different constructs, measurement reliability, restricted ranges, or nonspecific pathways. A predictive association between musical performance and cognitive change does not by itself demonstrate mediation. Prespecified longitudinal mediation or process models would be needed to test whether motor-skill acquisition, auditory learning, or other components of engagement account for cognitive transfer.

### 8.3. Individual and Social Moderators

Age, baseline dexterity, cognition, hearing, education, prior informal musical experience, motivation, and health can influence learning. Participants with lower baseline performance sometimes show larger gains, but regression to the mean and greater room for improvement are competing explanations. For example, the Train the Brain with Music cognitive-flexibility analysis indicated greater improvement among participants with lower baseline cognitive-flexibility performance, but such moderation should not be interpreted as evidence that lower-functioning individuals necessarily benefit more across domains [28]. Self-selected continuation studies are especially vulnerable to selection, motivation, and health-related differences between those who continue and those who discontinue practice.

Group instruction adds social coordination, observation, accountability, and ensemble timing. These features may enhance adherence and create meaningful interpersonal motor demands. They also complicate attribution when passive or no-treatment controls are used. Active-control groups should match contact, expectancy, group structure, and musical exposure while differing in the motor feature of interest. Where feasible, objective practice capture through MIDI, sensorized instruments, or time-stamped software should supplement self-report and allow analysis of dose, variability, and spacing. Such monitoring would help distinguish prescribed dose from actual practice exposure and permit testing of whether the amount and temporal distribution of practice predict motor, cognitive, or neural change.

## 9. Methodological Priorities for Future Research

The current evidence base remains limited by modest sample sizes, heterogeneous comparators and intervention protocols, repeated publications from a relatively small number of parent cohorts, and inconsistent outcome selection. The current evidence base is concentrated largely on piano, while string instruments, conventional woodwind and brass modalities, and lower-limb-intensive percussion remain sparsely tested. Practice dose is often prescribed or self-reported rather than measured objectively, and retention, motor transfer, and everyday function are seldom assessed. Because this is a narrative review, study selection and interpretive weighting also remain judgment-based, and no formal risk-of-bias or certainty assessment was performed.

Future studies should prioritize adequately powered randomized controlled designs with prespecified primary outcomes and active comparators matched for instructor contact, musical exposure, expectancy, and social or cognitive engagement. Direct comparisons between instrumental modalities would be particularly informative when they manipulate a clearly defined motor property, such as fine versus gross action, symmetric versus asymmetric bimanual coordination, hand-only versus hand–foot coordination, or continuous respiratory control, while holding teaching dose and musical content as constant as feasible. Reports arising from shared parent cohorts should also be identified explicitly to avoid overstating independent replication.

Outcome selection should be linked to the specific demands of the intervention. Behavioral assessment should include standardized measures of manual dexterity, finger individuation, speed–accuracy trade-offs, bimanual coordination, synchronization–continuation timing, and, where appropriate, gait, balance, postural control, or interlimb coordination. Musical performance should be quantified objectively to verify skill acquisition. Neural measures, including structural MRI, diffusion imaging, functional MRI, EEG, and MEG, should be selected according to prespecified mechanisms and interpreted with clear distinctions among structural increase, preservation, activation reduction, connectivity change, and oscillatory modulation.

Transfer should be operationalized explicitly. Task-specific learning should be distinguished from near transfer to structurally similar but untrained tasks and from far transfer to broader cognitive or everyday outcomes. Near-transfer measures should share clearly defined processes with the trained activity while remaining independent of the practiced repertoire, whereas far-transfer outcomes should be selected only when a plausible mechanistic pathway is specified in advance. Retention should be assessed after a no-training interval to distinguish temporary performance effects from durable learning.

Training exposure should be characterized in terms of duration, frequency, session length, home practice, progression, task difficulty, and actual practice dose. MIDI logs, instrument sensors, software timestamps, or recorded practice samples may provide more objective estimates of exposure, spacing, tempo, accuracy, and progression than self-report alone. Dose–response studies should manipulate exposure prospectively, and controlled late-life beginner studies are particularly needed for strings, conventional woodwind and brass instruments, and percussion protocols that explicitly quantify lower-limb and interlimb demands.

## 10. Conclusions

Musical instrument practice is a complex sensorimotor activity and a form of motor–cognitive skill training. In healthy older adults, the most defensible motor findings are improved task-specific bimanual synchronization, the acquisition and retention of musical actions, selective near transfer to internally maintained timing, and, in the largest long-term active-control cohort, improvement in untrained fine-motor control after one year of piano practice. Evidence for generalized dexterity, broad rhythmic transfer, interlimb coordination, gait, balance, and everyday motor function remains limited or mixed.

Cognitive effects are selective. Processing speed, verbal fluency or switching, and some working-memory outcomes improve in several studies, but null results are common, and benefits often occur in active comparison groups. Neuroplastic findings show that musical learning in later life can be accompanied by measurable structural and functional brain changes, yet cortical increase, structural preservation, reduced activation, altered connectivity, and oscillatory modulation must not be conflated. Few studies have directly tested whether neural change mediates motor or cognitive benefit.

Instrumental practice can provide an engaging and adaptable form of sensorimotor and motor–cognitive skill practice that integrates repeated movement with auditory feedback, cognitive control, and progressive learning. The next generation of research should measure the motor learning that the intervention is presumed to deliver, test retention and transfer, compare instrument-specific demands, include standardized motor outcomes, capture practice objectively, and account explicitly for parent-cohort dependence. On current evidence, the field supports cautious optimism about targeted sensorimotor and cognitive adaptation, not broad claims of neuroprotection or dementia prevention.

## Figures and Tables

**Table 1:** Characteristics of core intervention studies contributing to the narrative synthesis.

Study Report(s)	Design and Sample	Instrument and Training	Dose and Comparator	Motor and Sensorimotor Outcomes	Cognitive and Neuroplastic Outcomes	Main Finding and Interpretive Boundary
James et al. [20]; Worschech et al. [8,21,22,23,24]; Jünemann et al. [14,25]; Marie et al. [26]; Losch et al. [27]; Mack et al. [28]	Train the Brain with Music parent RCT, Germany and Switzerland; *N* = 156 randomized; healthy older adults, approximately 64–78 years across reports; minimal previous musical experience	Piano training with progressively graded repertoire and technique	Weekly 60 min supervised lesson plus approximately 30 min/day assigned home practice for 12 months; active music-culture comparator involving listening activities matched for contact and homework	Musical skill acquisition; untrained unimanual fine-motor control; individual motor trajectories	Both groups improved on several cognitive measures; piano showed a greater improvement in number-switch mixing cost after 12 months, while change in pianistic skill did not explain change in cognitive flexibility; bilateral auditory cortical-thickness increase; fornix fiber density stable in piano but declined in control; relative preservation of right auditory-cortex gray matter; altered auditory-stream connectivity; fine-motor change coupled with contralateral M1 gray-matter change	Largest multimodal active-control cohort. Piano-specific effects must be distinguished from changes shared by both musical conditions and from relative preservation. Multiple reports derive from the same parent sample.
Bugos [6]	Matched three-arm study, USA; 180 assigned, 135 completed; final piano *n* = 49, percussion *n* = 38, listening *n* = 48; age 60–80 years	Piano versus mallet percussion versus active music listening for 16 weeks	Weekly 45 min class; approximately 30 min/day or 3 h/week logged home practice	Bimanual coordination, synchronization, rhythmic-pattern performance, finger tapping	Selected gains in working memory and visual scanning; no clear response-inhibition advantage	Both active motor curricula changed trained-domain coordination; piano produced the largest hand-synchronization gain. Nonrandom allocation and similarity between training and outcome constrain generalization.
Bugos and Wang [29]	Three-arm RCT, USA; *N* = 155; healthy novices aged 60–80 years	Group piano versus computerized cognitive training versus no-treatment control	Two 90 min sessions/week for 16 weeks; no outside piano practice	No standardized motor-transfer endpoint	Both active groups improved working memory and processing speed; piano showed a specific category-switching fluency advantage; several outcomes were null	Supports selective rather than global cognitive transfer; does not establish generalized motor transfer.
Bugos and Kochar [30]	Within-participant pilot study, USA; *N* = 34; mean age 70.79 years; older piano novices	Intensive individual and group piano curriculum	30 h over 2 weeks; 3 h/day on 10 training days; within-participant no-training lead-in	No standardized motor-transfer endpoint reported	Processing speed and verbal fluency improved; verbal memory did not	Concentrated pilot evidence. The within-participant no-training period does not fully control expectancy, repeated testing, or instructor contact.
Bugos and Cooper [31]	Controlled pilot, USA; *N* = 20; mean age 73.15 years	Mallet-percussion instruction	Eight 2 h sessions; active autobiographical-recall discussion comparator	No standardized motor-transfer endpoint	Improved music self-efficacy; only a trend toward better Trail Making Test Part B performance	Small pilot; cognitive evidence was not definitive and generalized motor transfer was not assessed.
Santos et al. [32]	Randomized active-comparator study, Brazil; 31 healthy adults aged ≥60 years randomized; 28 completed, percussion/improvisation *n* = 15 and choir *n* = 13; mean age 68.40 and 67.31 years, respectively	Percussion-based musical improvisation using multiple easy-to-handle percussion instruments	Eight weekly 60 min sessions; active choir comparator matched for intervention duration and setting	Timed Up and Go and bilateral Finger-Tapping Test; no significant intervention-related effects on mobility or finger-tapping performance	Clock Drawing Test showed a significant group-by-time interaction favoring the improvisation condition; Trail Making Test Part A improved across groups, whereas verbal fluency, memory, and the other executive measures did not show intervention-specific benefits	The active musical comparator and blinded outcome assessment strengthen specificity testing, but the sample was small and the intervention lasted only eight weeks. Importantly, direct motor outcomes were null, showing that motor involvement in percussion training does not by itself establish generalized motor transfer.
Bugos et al. [33]	Randomized jazz-piano pilot, USA; final *N* = 37, training *n* = 17 and control *n* = 20; age 60–85 years	Intensive jazz-piano training	30 h over 2 weeks; passive no-training comparator	Musical skill; no standardized manual-transfer endpoint	Improved PASAT and Stroop performance; frontal theta maintained in piano participants while declining in controls; parietal alpha showed a group-by-time interaction	Preliminary behavioral and EEG evidence. Maintenance and interaction effects should not be interpreted as generalized neural enhancement.
Fujioka and Ross [13]	Nonrandomized controlled study; *N* = 14, piano *n* = 7 and no-training *n* = 7; mean age 63.5 years; healthy older adults	Individual piano training	Fifteen individualized lessons over approximately one month; passive no-training comparator	Synchronization–continuation finger tapping; continuation-phase timing stability improved, whereas synchronization did not show a comparable training benefit	MEG during passive metronome listening showed an increased range of beat-induced beta-band modulation after training	Provides rare evidence of near transfer from piano practice to internally maintained timing, accompanied by altered beta-band modulation. The very small sample, nonrandom assignment, and passive comparator limit causal precision; the neural change accompanied behavioral improvement, but mediation was not tested.
MacRitchie et al. [34]	Randomized wait-list crossover study, Australia; *N* = 15 completed; mean age 70.9 years	Small-group piano instruction	One 60 min class/week for 10 weeks; approximately 30 min/day recommended home practice; inactive wait-list comparator before crossover	Jebsen Taylor Hand Function Test and visuomotor synchronization task; no clear training-related improvement on either measure	Moderate evidence for improvement in Trail Making Test Part A; moderate evidence for negative training effects on Part B and the B–A difference score	Very small sample. Direct motor measures did not show the hypothesized improvements; the Part A finding is more consistent with a mixed visuomotor and processing-speed effect than with generalized manual-dexterity transfer.
Guo et al. [9]	Keyboard-harmonica RCT, Japan; 53 completed; training *n* = 27, control *n* = 26; mean age approximately 73 years	Simplified right-hand keyboard-harmonica training	Weekly 60 min lesson plus assigned home practice for 4 months; passive control	No standardized manual or respiratory-transfer outcome	Improved delayed story recall; reduced right SMA, precuneus, and posterior-cingulate activation and altered functional connectivity	Reduced activation was interpreted as potentially reflecting greater neural efficiency, but this interpretation remains inferential.
Wang et al. [35]; Kakihara et al. [36]	Ten-week melodica parent pseudo-randomized wait-list controlled trial, Japan; 60 healthy older adults analyzed; age 61–87 years	Simplified melodica instruction	Ten weekly 70 min group sessions plus diary-monitored home practice; wait-list usual-activity control	Music-performance rating; paced-continuation timing showed no intervention effect	Phonological verbal fluency improved; working-memory response-time evidence was marginal; later analysis reported an executive composite advantage	Early musical performance predicted cognitive change but was not tested as a causal mediator. Both reports derive from the same parent sample.
Chmiel et al. [12]; MacRitchie et al. [37]	Active Minds Music Ensemble longitudinal cohort, Australia; *N* = 68 healthy novices aged 65–79 years	Twelve-month program alternating digital piano and iPad-based virtual keyboard	One 60 min lesson every 2 weeks; approximately 30 min/day recommended practice; no external comparator	Melodic learning and retention; dual-finger and maximal single-finger tapping	Musical-learning trajectories predicted selected cognitive-transfer measures	Objective skill acquisition and retention were demonstrated; dual-finger but not maximal single-finger tapping improved. Absence of an external control limits causal inference.
Shinada et al. [10]	Open-label randomized trial, Japan; 27 entered baseline, 23 completed; age 65–74 years	Participant-selected keyboard, electronic drums, or bass guitar in group band sessions	One 90 min session/week for 16 weeks; home practice prohibited; passive control	Instrumental performance practiced; gait, balance, dexterity, and interlimb transfer not assessed	MMSE total and delayed logical-memory scores improved; executive-function outcomes did not	Small heterogeneous-modality study. Cognitive findings cannot be attributed to a specific instrument or motor demand.

*Note.* This table includes intervention studies forming the core contemporary evidence base in healthy or cognitively unimpaired older adults. Multiple publications derived from the same parent trial or cohort are grouped within a single row and should not be interpreted as independent replications. James et al. [20] identify the Train the Brain with Music parent-trial protocol, and it is not treated as independent outcome evidence. “No standardized motor-transfer endpoint” indicates that the cited report did not assess an independent standardized motor outcome beyond instrumental or task-specific performance; it does not indicate a null motor effect. EEG = electroencephalography; MEG = magnetoencephalography; M1 = primary motor cortex; MMSE = Mini-Mental State Examination; PASAT = Paced Auditory Serial Addition Test; RCT = randomized controlled trial; SMA = supplementary motor area.

**Table 2:** Motor and sensorimotor demands of instrument modalities represented in the reviewed evidence and major evidence gaps.

Modality	Effector Organization	Fine Motor and Finger Individuation	Bimanual and Interlimb Coordination	Visuomotor Demands	Auditory–Motor and Sensory Feedback	Respiratory and Postural Demands	Principal Outcomes Investigated	Evidence Boundary, Major Gap(s), and Source
**Piano and electronic keyboard**	Two-hand distal actions; protocols ranged from relatively symmetric exercises to differentiated hand roles	Individuated finger sequencing, temporal precision, and graded keypress; force demands vary according to acoustic, digital, or simplified keyboard configuration	Substantial bimanual demand in most curricula; lower-limb involvement was limited or not a focus of the reviewed protocols	Notation-to-key mapping, visual search, and hand-position monitoring	Immediate pitch and timing feedback supporting prediction, online monitoring, and error correction	Predominantly seated trunk and upper-limb posture; minimal cardiorespiratory or respiratory load	Bimanual synchronization, unimanual fine-motor control, tapping, continuation-phase timing, beat-related beta modulation, musical skill acquisition and retention, executive outcomes, and structural, functional, and electrophysiological neural adaptations	Bugos [6]; Bugos and Kochar [30]; Bugos and Wang [29]; Fujioka and Ross [13]; MacRitchie et al. [34]; Jünemann et al. [14,25]; Losch et al. [27]; Mack et al. [28]; Marie et al. [26]; Worschech et al. [8,21,22,23,24]. Piano is the best represented modality, but curricula, dose, comparator, and outcome selection are heterogeneous.
**Jazz piano**	Two-hand key actions combined with improvisatory sequence generation	Distal sequencing and temporal precision; task-specific demands depend on voicing, chordal structure, and improvisation syllabus	Bimanual coordination with potentially differentiated rhythmic and harmonic roles	Chord and scale mapping; score dependence may be reduced when improvisation predominates	Continuous auditory feedback and online evaluation of self-generated musical material	Predominantly seated upper-limb posture; minimal cardiorespiratory demand	PASAT, Stroop performance, frontal theta, parietal alpha, and musical performance	Bugos et al. [33]. Intensive pilot with a small sample and passive comparator; no standardized manual-transfer endpoint.
**Mallet percussion and percussion-based improvisation**	Bilateral striking involving distal and proximal components, spatial targeting, and rebound control; percussion-based improvisation also involved multiple instruments and less standardized action patterns	Precise grip and strike timing, with larger movement amplitudes than typical keyboard actions; manual demands varied more widely in the improvisation protocol	Substantial bilateral coordination in mallet-based curricula; bilateral demands were more heterogeneous in percussion-based improvisation	Spatial layout of targets and continuous mallet-to-target mapping	Salient impact onset provides immediate temporal and spatial error feedback	Seated or standing depending on protocol; upper-body involvement is substantial but physiological load was not quantified	Bimanual coordination, synchronization, rhythmic-pattern performance, working memory, visual scanning, music self-efficacy, finger tapping, functional mobility, and selected executive outcomes	Bugos [6]; Bugos and Cooper [31]; Santos et al. [32]. The improvisation intervention used heterogeneous percussion instruments and did not improve finger tapping or Timed Up and Go performance relative to choir training. Findings should therefore not be generalized across percussion modalities or interpreted as evidence of generalized motor transfer.
**Electronic drums and mixed band**	Repetitive arm striking with possible foot-pedal actions; mixed ensembles also included keyboard or bass guitar	Depends on the selected instrument; drum grip and strike timing do not necessarily require the degree of finger individuation typical of keyboard performance	Potentially substantial hand–hand and hand–foot coordination, depending on the instrument selected	Spatial mapping to drum pads, keyboard, or fretboard	Strong rhythmic feedback combined with continuous ensemble-based auditory information	Potential seated postural and lower-limb involvement; these demands were not directly quantified	Global cognition, delayed story memory, and mood; gait, balance, dexterity, and interlimb transfer were not assessed	Shinada et al. [10]. Instrument choice was heterogeneous and outcomes were not stratified according to motor demands, limiting instrument-specific interpretation.
**Melodica and keyboard harmonica**	Reviewed protocols used predominantly right-hand key selection combined with blowing while the other hand supported the instrument	One-hand finger sequencing and timing; less manual complexity than the reviewed two-handed piano curricula	Limited bimanual demand in the reviewed protocols; the contralateral hand primarily supported the instrument	Key-location mapping using simplified notation or visual cues	Auditory pitch and timing feedback combined with oral airflow-related feedback	Respiratory coordination is inherent to performance, but embouchure, ventilatory, and pulmonary outcomes were not directly assessed	Verbal memory, verbal fluency, executive outcomes, task-related activation, functional connectivity, long-term putamen findings, oral diadochokinesis, grip strength, and skeletal muscle mass	Guo et al. [9]; Mizutani et al. [52]; Wang et al. [19,35]; Kakihara et al. [36]. Findings derive predominantly from simplified one-hand protocols and should not be generalized to conventional wind instruments. The Mizutani et al. observational study found a small increase in lip oral diadochokinesis, whereas grip strength and skeletal muscle mass declined; pulmonary function was not assessed.
**Touchscreen virtual keyboard**	Digit selection on a flat touchscreen with minimal key travel and mechanical resistance	Lower force and proprioceptive key-travel demands than a physical keyboard, while spatial and temporal digit selection remain necessary	Can involve single-finger and dual-finger patterns; bilateral demands depend on the programmed task	Strong visual–spatial mapping to the touchscreen interface	Immediate synthesized auditory feedback with reduced tactile and mechanical landmarking compared with a physical keyboard	Predominantly seated upper-limb posture; minimal respiratory demand	Melodic learning and retention, dual-finger and maximal single-finger tapping, and cognitive-transfer predictors	Chmiel et al. [12]; MacRitchie et al. [37]. Instrument and task order were randomized and counterbalanced within an uncontrolled crossover curriculum; the absence of an external comparator limits causal inference.
**String instruments**	Bowed strings require asymmetric left-hand fingering and right-hand bow control; plucked-string demands vary according to technique	Potentially substantial left-hand finger individuation, positional precision, and force control, with qualitatively different demands on the right hand	Substantial asymmetric bimanual coordination	Continuous fingerboard and bow-position mapping; visual dependence may decrease with expertise	Pitch and timbre are continuously coupled to finger position and bowing or plucking actions	Instrument-specific shoulder, upper-limb, trunk, and seated or standing postural demands	No eligible late-life beginner intervention identified; expert studies provide evidence of instrument-specific sensorimotor organization	Elbert et al. [38]. Mechanistic expert evidence illustrates long-term sensorimotor specialization but cannot establish effects of beginning string training in older age.
**Recorder and conventional woodwind/brass instruments**	Two-hand fingering may interact with embouchure, tongue articulation, and breath timing; specific organization differs substantially across instrument families	Potentially substantial finger sequencing in many woodwinds; brass instruments rely less on extensive key arrays and more strongly on combined valve, embouchure, and airflow control	Variable bilateral hand roles combined with oral and respiratory coordination	Notation-to-fingering mapping and instrument-specific spatial organization	Continuous auditory pitch and timbre feedback linked to airflow, articulation, and finger or valve actions	Substantial respiratory and embouchure involvement; postural requirements vary by instrument	An uncontrolled recorder intervention assessed cognitive and musical-learning outcomes; no controlled healthy-only late-life beginner evidence was identified for generalized motor, respiratory, or neural transfer from conventional woodwind or brass training	MacAulay et al. [53]. Thirty-five older adults completed 12 weekly 1 h group recorder lessons, but the study had no control group and included cognitive performance ranging from normal to mild cognitive impairment; it is therefore contextual rather than core healthy-aging evidence. No controlled healthy-only late-life beginner intervention was identified for other conventional woodwind or brass modalities.
**Body percussion and nonpitched mixed music**	Whole-body or upper-limb strikes, claps, and actions on nonpitched instruments	Finger individuation is generally less central than in keyboard performance, although manual precision varies across tasks	Bilateral coordination and, in some activities, interpersonal synchronization	Formal notation may be limited; action observation, imitation, and spatial cueing may predominate	Rhythmic auditory feedback is combined with visual, proprioceptive, and social information	Potential gross-motor and postural involvement, but physiological load was not quantified	Stroop-related outcomes, auditory ERP measures, and selected cognitive outcomes; no isolated instrument-specific motor-transfer measure	Alain et al. [42]; Degé and Kerkovius [54]. Programs combined percussion with voice, singing, body movement, or other components, preventing attribution specifically to instrumental motor practice.

*Note.* Entries characterize the task demands of the exact study protocols and mechanistic sources cited in the final column and should not be interpreted as universal properties of every instrument within a family. Descriptions of motor, sensorimotor, respiratory, and postural demands do not imply that these functions improved unless they were directly assessed as outcomes. Studies derived from the same parent trial or cohort should not be interpreted as independent replications. EEG = electroencephalography; ERP = event-related potential; fMRI = functional magnetic resonance imaging; MEG = magnetoencephalography; MRI = magnetic resonance imaging; PASAT = Paced Auditory Serial Addition Test.

## Data Availability

No new data were created or analyzed in this study. Data sharing is not applicable to this article.

## Supplementary Materials

The following supporting information can be downloaded at https://www.mdpi.com/article/10.3390/brainsci16101038/s1: Table S1: Complete database-specific search strategies and retrieval counts; Table S2: Contextual, mixed-status, longitudinal follow-up, and observational studies informing the narrative synthesis.
