# Supplementary material for: Musical Instrument Practice as Sensorimotor and Motor–Cognitive Skill Training in Healthy Aging
Source: Brain Sci. 2026 Sep 28;16(10):1038. doi: 10.3390/brainsci16101038 (PMC13702461; doi:10.3390/brainsci16101038)
Supplement: Supplementary file 1 [file brainsci-16-01038-s001.zip › Supplementary_Table_S1.pdf]

Supplementary Table S1. Complete Database-Specific Search Strategies and Retrieval Counts.

Database	Last search date	Records retrieved	Exact search string
**PubMed/MEDLINE**	**31 August 2026**	**510**	(("musical instrument"[Title/Abstract] OR "musical instruments"[Title/Abstract] OR "instrumental practice"[Title/Abstract] OR "instrumental training"[Title/Abstract] OR "music training"[Title/Abstract] OR "musical training"[Title/Abstract] OR "instrument playing"[Title/Abstract] OR "instrument learning"[Title/Abstract] OR piano[Title/Abstract] OR keyboard[Title/Abstract] OR percussion[Title/Abstract] OR drum*[Title/Abstract] OR drumming[Title/Abstract] OR melodica[Title/Abstract] OR "keyboard harmonica"[Title/Abstract] OR "string instrument"[Title/Abstract] OR "string instruments"[Title/Abstract] OR violin*[Title/Abstract] OR viola[Title/Abstract] OR cello[Title/Abstract] OR guitar*[Title/Abstract] OR "wind instrument"[Title/Abstract] OR "wind instruments"[Title/Abstract] OR "brass instrument"[Title/Abstract] OR "brass instruments"[Title/Abstract] OR flute*[Title/Abstract] OR clarinet*[Title/Abstract] OR saxophone*[Title/Abstract] OR recorder*[Title/Abstract] OR trumpet*[Title/Abstract] OR trombone*[Title/Abstract] OR horn*[Title/Abstract] OR oboe*[Title/Abstract] OR bassoon*[Title/Abstract] OR harmonica[Title/Abstract] OR marimba*[Title/Abstract] OR vibraphone*[Title/Abstract] OR xylophone*[Title/Abstract] OR "drum set"[Title/Abstract] OR "snare drum"[Title/Abstract] OR timpani[Title/Abstract] OR cajon[Title/Abstract]) AND ("older adult"[Title/Abstract] OR "older adults"[Title/Abstract] OR elderly[Title/Abstract] OR aged[Title/Abstract] OR aging[Title/Abstract] OR ageing[Title/Abstract] OR "healthy aging"[Title/Abstract] OR "healthy ageing"[Title/Abstract] OR senior*[Title/Abstract] OR "older people"[Title/Abstract] OR "older person"[Title/Abstract] OR "older persons"[Title/Abstract] OR "Aged"[MeSH Terms]) AND ("motor function"[Title/Abstract] OR "motor control"[Title/Abstract] OR "motor skill"[Title/Abstract] OR "motor skills"[Title/Abstract] OR "fine motor"[Title/Abstract] OR dexterity[Title/Abstract] OR "finger dexterity"[Title/Abstract] OR coordination[Title/Abstract] OR "bimanual coordination"[Title/Abstract] OR "interlimb coordination"[Title/Abstract] OR sensorimotor[Title/Abstract] OR "motor learning"[Title/Abstract] OR timing[Title/Abstract] OR synchronization[Title/Abstract] OR cognition[Title/Abstract] OR "executive function"[Title/Abstract] OR "executive functions"[Title/Abstract] OR "working memory"[Title/Abstract] OR inhibition[Title/Abstract] OR "cognitive flexibility"[Title/Abstract] OR "processing speed"[Title/Abstract] OR attention[Title/Abstract] OR "verbal fluency"[Title/Abstract] OR memory[Title/Abstract] OR neuroplasticity[Title/Abstract] OR "brain plasticity"[Title/Abstract] OR "functional connectivity"[Title/Abstract] OR "gray matter"[Title/Abstract] OR "grey matter"[Title/Abstract] OR "white matter"[Title/Abstract] OR "motor cortex"[Title/Abstract] OR "supplementary motor area"[Title/Abstract] OR cerebellum[Title/Abstract] OR MRI[Title/Abstract] OR fMRI[Title/Abstract] OR EEG[Title/Abstract])) AND ("2016/01/01"[Date - Publication] : "2026/08/31"[Date - Publication]) AND English[Language]

**Scopus**	**31 August 2026**	**1,274**	TITLE-ABS-KEY(("musical instrument" OR "musical instruments" OR "instrumental practice" OR "instrumental training" OR "music training" OR "musical training" OR "instrument playing" OR "instrument learning" OR piano OR keyboard OR percussion OR drum* OR drumming OR melodica OR "keyboard harmonica" OR "string instrument" OR "string instruments" OR violin* OR viola OR cello OR guitar* OR "wind instrument" OR "wind instruments" OR "brass instrument" OR "brass instruments" OR flute* OR clarinet* OR saxophone* OR recorder* OR trumpet* OR trombone* OR horn* OR oboe* OR bassoon* OR harmonica OR marimba* OR vibraphone* OR xylophone* OR "drum set" OR "snare drum" OR timpani OR cajon) AND ("older adult" OR "older adults" OR elderly OR aged OR aging OR ageing OR "healthy aging" OR "healthy ageing" OR senior* OR "older people" OR "older person" OR "older persons") AND ("motor function" OR "motor control" OR "motor skill" OR "motor skills" OR "fine motor" OR dexterity OR "finger dexterity" OR coordination OR "bimanual coordination" OR "interlimb coordination" OR sensorimotor OR "motor learning" OR timing OR synchronization OR cognition OR "executive function" OR "executive functions" OR "working memory" OR inhibition OR "cognitive flexibility" OR "processing speed" OR attention OR "verbal fluency" OR memory OR neuroplasticity OR "brain plasticity" OR "functional connectivity" OR "gray matter" OR "grey matter" OR "white matter" OR "motor cortex" OR "supplementary motor area" OR cerebellum OR MRI OR fMRI OR EEG)) AND PUBYEAR > 2015 AND PUBYEAR < 2027 AND (LIMIT-TO(LANGUAGE, "English")) AND (LIMIT-TO(DOCTYPE, "ar"))
**Web of Science Core Collection**	**31 August 2026**	**1,760**	TS=(("musical instrument" OR "musical instruments" OR "instrumental practice" OR "instrumental training" OR "music training" OR "musical training" OR "instrument playing" OR "instrument learning" OR piano OR keyboard OR percussion OR drum* OR drumming OR melodica OR "keyboard harmonica" OR "string instrument" OR "string instruments" OR violin* OR viola OR cello OR guitar* OR "wind instrument" OR "wind instruments" OR "brass instrument" OR "brass instruments" OR flute* OR clarinet* OR saxophone* OR recorder* OR trumpet* OR trombone* OR horn* OR oboe* OR bassoon* OR harmonica OR marimba* OR vibraphone* OR xylophone* OR "drum set" OR "snare drum" OR timpani OR cajon) AND ("older adult" OR "older adults" OR elderly OR aged OR aging OR ageing OR "healthy aging" OR "healthy ageing" OR senior* OR "older people" OR "older person" OR "older persons") AND ("motor function" OR "motor control" OR "motor skill" OR "motor skills" OR "fine motor" OR dexterity OR "finger dexterity" OR coordination OR "bimanual coordination" OR "interlimb coordination" OR sensorimotor OR "motor learning" OR timing OR synchronization OR cognition OR "executive function" OR "executive functions" OR "working memory" OR inhibition OR "cognitive flexibility" OR "processing speed" OR attention OR "verbal fluency" OR memory OR neuroplasticity OR "brain plasticity" OR "functional connectivity" OR "gray matter" OR "grey matter" OR "white matter" OR "motor cortex" OR "supplementary motor area" OR cerebellum OR MRI OR fMRI OR EEG)) AND PY=(2016-2026) AND LA=(English) AND DT=(Article)

Note. Searches were last updated on 31 August 2026 and covered the predefined contemporary evidence window beginning on 1 January 2016. The searches retrieved 510 records from PubMed/MEDLINE, 1,274 from Scopus, and 1,760 from Web of Science Core Collection, corresponding to 3,544 database records before between-database deduplication. PubMed/MEDLINE used an exact publication-date range, whereas Scopus and Web of Science Core Collection used publication-year limits covering 2016–2026, with 31 August 2026 serving as the practical upper retrieval cutoff. Scopus and Web of Science searches were restricted to English-language articles; the PubMed/MEDLINE search was restricted to English-

language records, with publication type subsequently considered during eligibility assessment. Eligible evidence was determined according to the criteria described in Section 2.3, and overlapping publications from shared parent trials or cohorts were reconciled during study selection and data organization. Google Scholar was used supplementarily for targeted searching and citation tracking rather than as a reproducible primary database query. Backward and forward citation chasing from eligible primary studies and relevant reviews was used to identify additional reports and selected pre-2016 seminal or mechanistic sources. Because this was a narrative review, no PRISMA flow diagram, formal risk-of-bias assessment, or certainty-of-evidence grading was undertaken. DT = document type; EEG = electroencephalography; fMRI = functional magnetic resonance imaging; LA = language; MeSH = Medical Subject Headings; MRI = magnetic resonance imaging; PY = publication year; TITLE-ABS-KEY = Scopus title, abstract, and keyword fields; TS = Web of Science topic field.
