# Supplementary material for: Musical Instrument Practice as Sensorimotor and Motor–Cognitive Skill Training in Healthy Aging
Source: Brain Sci. 2026 Sep 28;16(10):1038. doi: 10.3390/brainsci16101038 (PMC13702461; doi:10.3390/brainsci16101038)
Supplement: Supplementary file 1 [file brainsci-16-01038-s001.zip › Supplementary_Table_S2.pdf]

Supplementary Table S2. Contextual, Mixed-Status, Longitudinal Follow-Up, and Observational Studies Informing the Narrative Synthesis.

Study report(s)	Design and sample	Exposure or intervention	Outcomes relevant to this review	Role in the present synthesis	Main interpretive boundary
**Lister et al. (2023)**	Keys to Staying Sharp RCT, USA; N = 268; older adults with normal cognition or MCI	Piano versus active music-listening intervention for 20 weeks	Auditory processing, cognition, everyday function	Contextual active-comparator evidence	Piano showed no superiority on prespecified outcomes; mixed cognitive status places the sample outside the healthy-only core evidence.
**MacAulay et al. (2019)**	Uncontrolled longitudinal study, USA; 35 completers; mean age approximately 70 years, range 59–81 years; cognitive status ranged from normal performance to scores compatible with MCI	Twelve weekly 60-min group recorder lessons for older musical novices	Musical learning, global cognition, executive function, verbal fluency, and visual memory	Contextual uncontrolled wind-instrument evidence	The study demonstrates feasibility of late-life beginner recorder training and reported pre–post cognitive changes, but it lacked a control group and included a cognitively heterogeneous sample. It therefore cannot establish training-specific effects and is not part of the healthy-only core intervention evidence.
**West et al. (2017)**	Three-arm randomized neuroimaging trial, Canada; 48 recruited; final MRI groups were small; age 55–75 years	Piano versus 3-D video-game training versus passive control for 6 months	Cognition and structural MRI	Contextual neuroplastic evidence	Age range extends below the principal ≥60-year population and final groups were very small. Hippocampal gray-matter increase occurred in the video-game group rather than the piano group; piano findings were confined to other regional changes.
**Wang et al. (2025)**	Four-year observational continuation of the Kyoto keyboard-harmonica parent cohort, Japan; continued n = 13, discontinued n = 19	Voluntary continuation or discontinuation of instrumental practice after the original intervention	Verbal working memory, putamen gray matter, cerebellar activation/connectivity	Long-term follow-up evidence	Continued practice was self-selected rather than randomized; findings are vulnerable to attrition, motivation, health, and lifestyle differences.
**Mizutani et al. (2023)**	Nonrandomized observational comparison, Japan; 30 enrolled and 23 analyzed; keyboard-harmonica n = 10 and exercise n	Fifteen keyboard-harmonica or exercise classes over approximately 10 months, with optional home assignments	Oral diadochokinesis, grip strength, skeletal muscle mass, and other physical-function measures	Contextual oral and physical-function evidence	Lip oral diadochokinesis increased slightly in the keyboard-harmonica group, whereas grip strength and skeletal muscle mass declined. Small sample, nonrandomized self-selected

Study report(s)	Design and sample	Exposure or intervention	Outcomes relevant to this review	Role in the present synthesis	Main interpretive boundary
	= 13; community-dwelling adults aged ≥60 years				participation, COVID-19-related movement restrictions, and absence of a pulmonary-function outcome preclude attribution of a generalized physical or respiratory benefit to instrumental practice.
**Degé and Kerkovius (2018)**	Three-arm pilot, Germany; N = 24 older women	Fifteen-week combined drumming-and-singing program versus literature training and no-treatment controls	Working memory and selected cognitive outcomes	Contextual multicomponent music evidence	Drumming was combined with singing, so effects cannot be attributed specifically to instrumental motor practice.
**Alain et al. (2019); Bugos et al. (2022); Lu et al. (2022)**	Three-arm arts-training parent cohort, Canada; 60 recruited; analytic samples varied across reports, with N = 50 for the time–frequency EEG analyses	Music program using voice, body percussion, and nonpitched instruments versus visual-art and passive controls	Cognitive outcomes; auditory ERPs; auditory-evoked oscillatory activity; response-suppression-related theta activity and functional connectivity	Contextual multimodal arts evidence	Reports derive from the same parent cohort and should not be treated as independent replications. The music curriculum combined voice, body percussion, and non-pitched instruments and therefore does not isolate instrumental motor practice.
**Kim and Yoo (2020)**	Exploratory controlled trial, South Korea; N = 20 women aged ≥65 years	Rhythm–motor dual tasks combining walking or bimanual tapping with instrument playing, chanting, or singing	Dual-task gait, mobility, bimanual tapping, Trail Making Test Part B	Adjacent motor–cognitive evidence	Highly relevant mechanistically but not an isolated instrumental-learning intervention.
**Yamashita et al. (2022)**	Cross-sectional musician cohort, Japan; 30 older musicians and 30 nonmusicians	Lifelong/current heterogeneous instrumental practice	Finger tapping, executive function, cerebellar gray matter and cerebellar–hippocampal connectivity	Long-term expertise and neuroplastic context	Cross-sectional design cannot distinguish training from selection, reserve, health, lifestyle, or other lifelong differences.
**Böttcher et al. (2022)**	DELCODE propensity-matched cross-sectional cohort, Germany;	Life-course musical activity	Global cognition, working memory, executive function, language,	Observational cognitive and	Musical exposure was heterogeneous and matching cannot eliminate residual confounding or establish causality.

Study report(s)	Design and sample	Exposure or intervention	Outcomes relevant to this review	Role in the present synthesis	Main interpretive boundary
	70 musically active and 70 inactive older adults		visuospatial performance, gray matter	structural context	
**Chaddock-Heyman et al. (2021); Ai et al. (2022)**	Illinois Musical Activity and Brain Imaging parent cohort, USA; overlapping older-adult samples, with analytic N varying across reports	Lifetime musical experience including instrumental and singing components	Inferior-frontal/parahippocampal volume; insula functional connectivity; cognition	Observational neuroimaging context	Both articles derive from the same parent cohort and should not be treated as independent evidence. Exposure was not exclusively instrumental, and associations may reflect pre-existing differences.
**Romeiser et al. (2021)**	Wisconsin Longitudinal Study, USA; N = 5,718 assessed in later life	High-school and continuing-adult instrumental engagement	Episodic-memory level and subsequent change	Large life-course observational evidence	Musical engagement was associated with higher memory level but not with a slower subsequent rate of decline; causal interpretation is not warranted.
**Moussard et al. (2016)**	Cross-sectional lifelong-musician study, Canada; 17 older musicians and 17 matched nonmusicians	Long-term instrumental practice	Executive control and task-related ERP measures	Mechanistic expertise evidence	Cross-sectional expertise differences cannot demonstrate effects of beginning instrumental training in later life.

Note. This supplementary table includes studies that informed interpretation but were not treated as core intervention evidence because of mixed cognitive status, participant ages extending below the principal older-adult threshold, multicomponent interventions that did not isolate instrumental practice, uncontrolled interventions, nonrandomized long-term continuation, or observational/cross-sectional designs. Studies originating from the same parent cohort are grouped and should not be considered independent replications. ERP = event-related potential; MCI = mild cognitive impairment; MRI = magnetic resonance imaging; RCT = randomized controlled trial.
